# Tobacco use and readiness to treat tobacco users among primary healthcare professionals in Soweto

**DOI:** 10.4102/safp.v66i1.5996

**Published:** 2024-10-29

**Authors:** Ann N. Alagidede, Olufemi B. Omole

**Affiliations:** 1 Department of Family Medicine and Primary Care, Faculty of Health Sciences, School of Medicine, University of the Witwatersrand, Johannesburg, South Africa

**Keywords:** healthcare professionals, cessation treatments, readiness, tobacco use, tobacco, training, willingness

## Abstract

**Background:**

Despite its hampering influence on the willingness of healthcare professionals (HCPs) to implement tobacco cessation treatments, the tobacco use status of these professionals remains understudied in South Africa.

**Methods:**

This cross-sectional study, which sampled 444 HCPs, was conducted in five community health centres in Soweto. A self-administered questionnaire collected information on socio-demography, tobacco use, quit attempts and readiness to implement tobacco cessation treatments for their patients.

**Results:**

The mean age was 41 years. Most were female, 80% (*n* = 355); single, 54.1% (*n* = 240) and black professionals, 91.6% (*n* = 405). About 22% (*n* = 96) were ever-users of tobacco, 12.6% (*n* = 56) current users and 9% (*n* = 40) ex-users. About 56.6% (*n* = 30) of current users had contemplated quitting in the past year. Approximately 68% (*n* = 300) and 82.2% (*n* = 365) of respondents were ready and willing to implement tobacco cessation treatments, respectively. Only 32% (*n* = 143) of respondents had received any training on tobacco use and cessation treatments. There was no significant association between tobacco use and readiness to implement cessation treatments (*p* = 0.50).

**Conclusion:**

Tobacco use is prevalent among HCPs and does not influence the implementation of cessation treatments in South African primary health care. Although most reported readiness and willingness to quit tobacco use, more training is required in both formal education and continued professional development.

**Contribution:**

This study demonstrates the alarming rate of tobacco product use among primary health care professionals in South Africa. While there is a strong willingness to implement tobacco cessation treatments for their patients, most healthcare professionals still require training to enhance their self-efficacy.

## Introduction

Global tobacco smoking rates have steadily decreased over the last three decades, dropping from around 34.2% in 2000 to 23.0% in 2020.^1^ However, according to the 2021 World Health Organization (WHO) report, over 8 million people worldwide die from tobacco use and exposure every year, with most of these deaths being premature.^2^ Tobacco usage is the leading cause of death in Asia and Eastern Europe, with rates exceeding 100 deaths per 100 000 people.^3^ These figures surpass the combined mortality from malaria, human immunodeficiency virus and tuberculosis.^4^

Tobacco use is still prevalent in South Africa despite a progressive tobacco control policy and regulatory programme. The recent 2021 Global Adult Tobacco Survey (GATS) estimates that nearly 30% of South Africans use tobacco.^5^ This is concerning because tobacco use is a known risk factor for several of the country’s top 10 leading causes of mortality from noncommunicable illnesses, including hypertension, ischaemic heart disease, chronic obstructive pulmonary disease, malignancies and diabetes.^6^ Aside from the harmful health implications, the economic cost of smoking was estimated to be R42 billion in 2016, with R14.8bn spent on healthcare (hospitalisations and outpatient visits).^7^

According to the 2017 health budget for South Africa, 84% of the general population depends on public sector healthcare. More importantly, this population includes a high-risk population in the working class,^8^ as indicated in the 2023 WHO report.^9^ This gives healthcare professionals (HCPs) in the public healthcare sector ready access to the proportion of the general population who use tobacco, particularly at the primary healthcare (PHC) level. An efficient strategy to decrease tobacco-related morbidity and mortality would, therefore, be to ensure that primary health care providers act as good role models and advocates for anti-tobacco measures during the clinical encounter.

Furthermore, HCPs can positively influence tobacco users to quit; however, studies addressing their readiness and willingness to do this are yet to be explored in South African PHC. The readiness of HCPs to implement tobacco cessation treatments (TCTs) is described by a construct of how important these TCTs are to them and how confident they are in executing them.^10^ Their willingness to do so is defined by how eager they are to conduct them. Indeed, their knowledge of anti-tobacco measures may influence their readiness to perform TCT.^11^

As health experts and patient advocates, HCPs in PHC are uniquely placed to respond to the tobacco epidemic. By implementing evidence-based tobacco cessation guidelines, HCPs in PHC can effectively counsel their patients on smoking cessation and treat tobacco dependence in patients.^12^ The opposite has also been shown to be true elsewhere, as HCPs who use tobacco are less likely to be willing and/or ready to implement TCT.^13^ There are no recent data on the proportion of healthcare providers who use tobacco in South Africa. In addition, their willingness and readiness to intervene in their patients’ tobacco use have not been explored. A helpful starting point to achieve this will be to determine the prevalence of tobacco use among HCPs in PHC and determine how tobacco use influences their willingness to counsel their patients on tobacco cessation practices and implement treatments.

It is currently unclear how many HCPs worldwide use tobacco (smoked, smokeless or heated), especially in low- to middle-income countries where few studies have been conducted. A recent review estimated that between 2000 and 2014, around 21% of HCPs use tobacco,^14^ with family physicians having the highest smoking prevalence at 24%.^14^ However, rates vary by country, with some reporting lower percentages: Poland (7.8%)^15^ and Estonia (6.7%).^16^ The recent coronavirus disease 2019 (COVID-19) led to increased stress and burnout among HCPs, with a 2021 French study showing a 24% increase in nicotine dependence among HCPs after the lockdown. This suggests that HCPs may have turned to tobacco and other substances as a coping mechanism.^17^ Within South Africa, three studies,^18,19,20^ two in 2012 and the most recent in 2018, found that HCPs and health sciences students had smoking rates ranging from 8% to 17%. Also, according to a study by Senkubuge et al.,^19^ data from 2008 showed that 18.6% of medical students used alternative tobacco products, such as water pipes, while 3.1% used smokeless tobacco. These findings suggest that tobacco use is common among HCPs in South Africa and that a new population of users, beyond cigarette smokers, may have emerged. However, this habit undermines HCPs’ moral authority in the fight against tobacco use, and those who use tobacco products are less likely to intervene in their patients’ tobacco use.^12^ These South African studies^18,19,20^ revealed significant gaps in health professional students and HCPs’ formal and in-service training, respectively, as only a small proportion of both categories are well equipped to effectively assist tobacco users in quitting.

With brief advice interventions, HCPs can significantly improve patients’ chances of quitting tobacco, increasing cessation rates by up to 3% per episode and cumulatively up to 50% in a lifetime.^21^ A study conducted in Malta in 2021 examined the effectiveness of a nurse-led brief smoking cessation training programme for HCPs. The findings showed that after the training, there was a notable increase in the delivery of TCTs by physicians, nurses and other healthcare workers.^22^ However, other studies show that physicians who smoke are less likely to offer tobacco cessation advice.^23^ Hence, identifying factors influencing HCPs’ intervention in smokers is essential for improving tobacco control interventions. Also, a study in two communities in South Africa found that many smokers are willing to quit if their clinician advises but the health system’s support to achieve this is inadequate.^24^ Several reasons have been posited: According to Meijer et al.,^25^ PHC providers face several challenges in providing interventions for tobacco addiction, including limited time, misconceptions about success rates, inadequate knowledge or counselling skills and concerns about damaging the doctor-patient relationship. Some HCPs also regard smoking as a habit rather than a disease^25^ and are even more reluctant if they are tobacco users themselves. These factors intertwine, creating a complex landscape that can hinder HCPs’ readiness to provide tobacco cessation services.

On the other hand, the Meijer et al. study^25^ found that HCPs who are motivated, have positive attitudes and have received cessation training are more likely to help patients quit using tobacco. Also, HCPs who identify strongly as role models are more likely to carry out interventions. Among patients, those who prioritise quitting, maintain a positive relationship with their clinician and use referral systems are more likely to succeed.

In South Africa, there is a dearth of literature on tobacco use among HCPs and a lack of information on how this influences their readiness and willingness to implement TCTs in patients. Therefore, this study examines the prevalence of tobacco use among HCPs in five PHC facilities in Soweto, Johannesburg district, South Africa. It further assesses HCPs’ readiness and willingness to implement TCT in their patients.

## Research methods and design

### Study design and setting

This cross-sectional study was conducted at the five public community health centres (CHCs) in Soweto sub-district D, one of the sub-districts in the City of Johannesburg Metropolitan Municipality, Gauteng province. Soweto is a large urban community with 29 primary health care facilities, including five CHCs, 23 local and provincial clinics and one district hospital. The services provided in the CHCs include well-baby care, immunisation, management of sexually transmitted infections, tuberculosis and other communicable diseases, noncommunicable chronic conditions, dental services, human immunodeficiency virus (HIV) and/or acquired immunodeficiency syndrome (AIDS) counselling, curative services for adults and children, and health education. These facilities cater to about 1.3m residents, who are mainly black South Africans.^26^ A cross-sectional study was selected because it was appropriate for determining prevalence at a point in time and the relationship between respondents’ tobacco use status and their readiness to implement tobacco cessation practices.^27^

### Study population

This study focussed on HCPs who provide clinical services, including counselling and have direct contact with patients in their facilities. The eligible HCPs include medical doctors, dentists, nurses and community health workers (CHWs) who are 18 years or older and work in CHCs in Soweto. The study excluded professional categories with limited or no patient contact as they are unlikely to influence patients’ tobacco use behaviour. A total of 480 HCPs were eligible for the study in the five CHCs.

### Sample size and sampling

With an eligible population of 480 HCPs, an acceptable margin of error of 5%, a 95% confidence level and a response distribution of 50%, the required sample size was estimated to be 214 using the Raosoft calculator.^28^ However, the researcher enrolled all eligible HCPs in the five CHCs in the district to account for possible refusal and missing and incomplete information.

### Data collection

The data collection tool was a questionnaire adapted from the key tobacco questions in the Global Adult Tobacco Surveillance Survey (GATSS).^29^ The WHO and Centers for Disease Control and Prevention formulated and used this questionnaire for tobacco surveillance and monitoring activities. The questionnaire is available from the WHO’s website at https://www.who.int/publications/i/item/9789241500951.^29^ This adapted questionnaire collected information on respondents’ socio-demographic characteristics, tobacco use patterns, quit attempts and readiness to provide TCTs.

### Measures

- Socio-demographic characteristics included age, gender, marital status, ethnicity, years of experience and professional category. Tobacco use patterns include duration, quantity and types of current use, past daily use for less than current daily smokers and past usage for current non-smokers, second-hand smoke exposure at home or in public places in the last month and concurrent use of other non-tobacco substances such as alcohol and marijuana. The GATSS description was used as stated for the tobacco use measures:
  ■ Current tobacco user: An HCP who uses tobacco products daily or occasionally.^29^
  ■ Ex-tobacco user: An HCP who has not used tobacco products within the last 12 months of this study’s commencement.^29^
  ■ Never tobacco user: An HCP who has never used tobacco.^29^
- Exposure to second-hand smoke was defined as HCPs who do not smoke but reported smoking occurring in their home or have been exposed to tobacco smoke in the workplace within the last 30 days.^29^
- Quit attempts made by respondents in the last year: This study categorised a quit attempt as an affirmative response ‘yes’ to the question, ‘Have you ever attempted to quit tobacco use?’ or if the participant reported trying to quit within the past year. The definition was intentionally crafted to avoid bias by not requiring a specific duration of abstinence, which would exclude those with the least likelihood of quitting. Instead, the focus was on the participant’s perception of making a serious effort to cease tobacco use permanently.^30^
- Respondents’ readiness to implement TCT (such as brief advice, counselling, pharmacotherapy, etc.) in their patients: The study defined respondents’ readiness as a combined construct of ‘their belief in the importance of offering tobacco cessation counselling and their confidence in being able to carry out this intervention(s) efficaciously’.^10^ This construct was operationalised using a five-point Likert scale for three questions (one to assess respondents’ perception of the importance of implementing TCT; the second to assess respondents’ confidence in being able to implement TCT and the third to assess their willingness to implement TCT). Two ordinal scales from 0 to 10 were combined to determine respondents’ readiness to implement TCTs: the importance ruler and the confidence ruler.^31^ Respondents who scored six and above on both rulers were considered ready, and those who scored five or less on one or both were classified as ‘not ready’ to offer tobacco use cessation interventions. A similar ordinal scale from 0 to 10 was used to measure the willingness of respondents to implement TCTs.^31^

Data collection occurred as follows: After ethics clearance and permissions were obtained from the Human Research and Ethics Committee and the district management, respectively, each facility manager was approached and asked for permission to access the PHC facility to recruit study respondents. All eligible HCPs were consecutively approached to enrol for recruitment into the study through the office of the Facility manager and the Heads of clinical units in each of the five CHCs in Soweto. Recruitment continued over 20 weeks (with two reminders or visits) until all eligible HCPs were approached. At the weekly meetings, each of the Heads of units (medical, nursing and CHWs) issued each eligible HCP in their category that showed interest a copy of the participant information leaflet detailing the study’s nature and purpose and a self-administered questionnaire. Questionnaires were completed at the convenience of the HCPs. Eligible HCPs who were absent during the meetings/researcher’s visits but who expressed interest and gave their email addresses had an electronic copy of the participant information leaflet and questionnaire sent to them by email. These made up 22 HCPs. At the end of each day, the investigator securely collected completed questionnaires.

### Pilot study

To ensure the validity of the measurement tool, the self-administered questionnaires were piloted on a sample of 40 HCPs (20 nurse clinicians, 10 doctors and 10 CHWs) at Hillbrow Community Health Centre, Johannesburg – a facility in another sub-district. Following piloting, some questions were revised for clarity: Firstly, the word ‘unsure’ was removed from the ordinal scales (rulers), which made it easier for the respondents to rate the level of importance, confidence and willingness. Secondly, question E32 in the GATSS was limited to only current tobacco users rather than all respondents, as the influence of their tobacco use on TCT can only be expressed by current users.

### Data management and analysis

The data from each patient’s questionnaire were captured on a secured Research Electronic Data Capture (REDCap) software platform and exported onto an Microsoft Excel spreadsheet. A statistician used the Statistical Package for Social Sciences (SPSS) statistical software 28 for the analyses.

Descriptive statistics were used to summarise respondents’ socio-demographic characteristics and determine the proportion of tobacco users. It also described respondents’ tobacco use patterns and the proportion of current users who had made a quit attempt. The analysis outcomes for categorical variables were expressed as frequencies and percentages. In contrast, the results for continuous variables were expressed as means with their standard deviations (s.d.) for normally distributed data and median with interquartile ranges (IQR) for skewed data.

Concerning the ordinal scales used to determine respondents’ readiness and willingness to implement TCTs, a one-sample *t*-test was applied to determine whether the average importance differed significantly from the central score of five. If the result was significant and the mean score was greater than five, the result was interpreted as significantly important. If the result was significant and the mean score was less than five, this was interpreted as significantly unimportant. Respondents who scored six and above on both rulers were considered ‘ready’, while those who scored five or less on one or both were classified as ‘not ready’ to implement TCTs. The same analysis applied to the willingness ruler. Regarding the ordinal scale for willingness, respondents who selected six or more on the willingness ruler were classified as willing to implement TCTs.

Using Chi-squared, *t*-tests or analyses of variance (ANOVA) as appropriate, comparisons and associations were conducted between respondents who were ‘ready’ and those who were ‘not ready’ regarding socio-demographic factors, tobacco use status and quit attempts. In all tests, statistical significance was deemed present when *p* was less than 0.05.

### Ethical considerations

The Human Research Ethics Committee (HREC Medical) of the University of the Witwatersrand granted ethical approval (clearance certificate number M210636). The Johannesburg Health District Research Committee (DRC) provided additional clearance (DRC Reference no.: 2020-11-017). All eligible respondents were given an information sheet prior to the study. This sheet explained the study’s objectives, scope, and potential risks. Participation was voluntary, as outlined in the participant information leaflet, and confidentiality was maintained through unique codes assigned to each participant. No identifying information was collected, and the initial list of eligible respondents was stored on a password-protected computer that was erased before data analysis. Only the researcher had access to the questionnaire responses. This study adhered to the 1964 Helsinki Declaration. The Ethics committee waived the requirement of written informed consent for participation, as completing the self-administered questionnaire was considered to imply consent to participate in the study.

## Results

Of the 480, a total of 447 respondents returned their questionnaires. Thirty respondents did not return the questionnaire despite reminders, while three returned it uncompleted and were excluded from the analysis. The remaining 444 constituted the study’s sample (Response rate of 92.5%).

### Socio-demographic characteristics of the study respondents

Table 1 summarises the socio-demographic profile of the study respondents. Most respondents were female, 80% (*n* = 355); single, 54.1% (*n* = 240) and black professionals, 91.6% (*n* = 405). The mean participant age was 41 years (s.d.: 11.0), and the median years of experience in healthcare were 12 years (IQR = 5–16).

**TABLE 1:** Socio-demographic characteristics of respondents.

Variable	Categories	Frequency (*n* = 444)	Percentage (%)
Age group of respondents (years)	19–29	79	17.8
	30–39	137	30.9
	40–49	105	23.6
	50–59	84	18.9
	60+	29	6.5
	Unspecified	10	2.3
Gender	Female	355	80.0
	Male	83	18.7
	Unspecified	6	1.4
Professional category	Medical practitioner	76	17.0
	Dental practitioner	12	2.7
	Nurse clinician	216	48.3
	Community health worker	140	31.3
Ethnicity	Black people	405	91.6
	Mixed race people	3	0.7
	Indian people	17	3.8
	White people	17	3.8
	Unspecified	2	0.4
Marital status	Divorced	18	4.1
	Single	240	54.1
	Married	137	30.9
	Widowed	19	4.3
	Separated	5	1.1
	Cohabiting	24	5.4
	Unspecified	1	0.2
Years of experience	1–5	117	26.4
	6–10	100	22.5
	11–15	97	21.8
	16–20	44	9.9
	21+	67	15.1
	Unspecified	19	4.3

### Tobacco use among healthcare providers

The prevalence of ever-use of tobacco among healthcare workers was 21.6%, composed of 8.9% (*n* = 40) ex-users and 12.5% (*n* = 56) current users. Among the current tobacco users, 64.3% (*n* = 36) smoked only cigarettes, 17.9% (*n* = 10) used only snuff and 14.3% (*n* = 8) used other forms of tobacco. The remainder 3.6% (*n* = 2) smoked cigarettes with snuff use or other products. Males were significantly more likely to report having used tobacco than their female counterparts (44.6% vs. 16.6% *p* < 0.001). All current snuff users were female, 100% (*n* = 11), while 98.8% (*n* = 82) of male HCPs had never used snuff, the difference being statistically insignificant (*p* = 0.29).

Tobacco use varied by professional category, with the highest prevalence among CHWs (35.7%). In bivariate analysis, more nurses had never smoked cigarettes, while doctors and dentists tended to report being ex-cigarette smokers and many CHWs were current smokers (*p* < 0.001; see Table 2).

**TABLE 2:** Prevalence of tobacco use among respondents.

Tobacco use	Response category	Professional category								Total (*n*)		Pearson’s chi-square/Fisher’s exact	
		Doctor		Dentist		Nurse		Community health worker				Test statistic	*p*-value
		Frequency (*n*)†	%†	Frequency (*n*)†	%†	Frequency (*n*)†	%†	Frequency (*n*)†	%†	Frequency (*n*)†	%†		
Cigarettes	Never	64	84.2	10	83.3	196	90.7	102	72.9	372	83.8	42.4	< 0.001
	Ex-user	9	11.8	2	16.7	14	6.5	9	6.4	34	7.7	-	-
	Current user	3	3.9	0	0.0	6	2.8	29	20.7	38	8.5	-	-
	Total	76	100.0	12	100.0	216	100.0	140	100.0	444	100.0	-	-
Snuff	Never	76	100.0	12	100.0	211	97.7	127	90.7	426	95.9	12.0	0.036
	Ex-user	0	0.0	0	0.0	2	0.9	5	3.6	7	1.6	-	-
	Current user	0	0.0	0	0.0	3	0.9	8	5.7	11	2.5	-	-
	Total	76	100.0	12	100.0	216	100.0	140	100.0	444	100.0	-	-
Other forms of tobacco	Never	71	93.4	10	83.3	212	98.1	139	99.3	432	97.3	19.4	0.001
	Ex-user	0	0.0	2	16.7	1	0.5	0	0	3	0.7	-	-
	Current user	5	6.6	0	0.0	3	1.4	1	0.7	9	2.0	-	-
	Total	76	100	12	100.0	216	100.0	140	100.0	444	100.0	-	-

† , Within professional category.

Among current tobacco users, many preferred mentholated manufactured cigarettes, 43% (*n* = 24). None reported using cigars, pipes or chewed tobacco leaves (Table 3).

**TABLE 3:** Tobacco use patterns among respondents.

Item	*n*	%	Mean	s.d.
**Type of tobacco and average daily use**				
Cigarette (number of cigarettes per day)	-	-	4.95	3.0
Snuff (number of dips per day)	-	-	2.38	1.7
Chewed tobacco leaves (number of times chewed per day)	-	-	0.75	1.5
E-cigarette (number of vapes per day)	-	-	0.75	1.0
**Patterns of tobacco use among current tobacco users**				
Manufactured cigarette – Non-menthol	16	29.0	-	-
Manufactured cigarette – Mentholated	24	43.0	-	-
Hand-rolled cigarette	1	2.0	-	-
Pipe-tobacco	-	-	-	-
Cigar	-	-	-	-
Traditional snuff	15	27.0	-	-
Industrially prepared snuff	2	4.0	-	-
Water-pipe/hookah/Hubble Bubble/Shisha	9	16.0	-	-
Chewed tobacco leaves	-	-	-	-
E-cigarette	3	5.0	-	-
Other	-	-	-	-
**Reasons for initiating tobacco use among current tobacco users**				
Peer pressure	12	21.0	-	-
Traditional practices	9	16.0	-	-
My parents used tobacco	3	5.0	-	-
Pressures of school work	6	11.0	-	-
Relationship pressures	6	11.0	-	-
Work pressures	6	11.0	-	-
I do not know	15	27.0	-	-
It helped my mental health state	8	14.0	-	-
Other	4	7.0	-	-
**Areas of tobacco use (*n* = 56)**				
Home	12	31.6	-	-
Work	5	13.2	-	-
Home and work	17	44.7	-	-
Designated smoking areas	1	2.6	-	-
Recreational places	2	5.3	-	-
Any place where they need to	10	26.3	-	-
**Conjoint tobacco and other recreationa l products use (*n* = 56)**				
Marijuana	7	12.5	-	-
Alcohol	32	57.1	-	-
Nyaope	1	1.8	-	-
Methamphetamine/tick	-	-	-	-
Other	-	-	-	-
**Second-hand smoke exposure (*n* = 142)**				
Home	46	32.4	-	-
Work	33	23.2	-	-
Both home and work	47	33.1	-	-
Other places	12	8.5	-	-

s.d., standard deviations.

The mean age of initiating tobacco use was 24 years (s.d. = 9.9). Among current tobacco users, cigarette smokers had a mean duration of smoking of 10.9 years (s.d. = 7.3), smoked an average of five cigarettes per day (s.d. = 3.0) and averaged three-pack years of smoking (s.d. = 3.1). The mean duration of snuff use was 8 years (s.d. = 8.9), with an average of three dips per day.

The majority of current cigarette smokers, 44.7% (*n* = 17), reported smoking both at their workplace and at home. About 31.6% (*n* = 12) smoke at home only, and 13.2% (*n* = 5) smoke exclusively at work (Table 3).

With regard to other substances used with tobacco, 57% (*n* = 32) drank alcohol while using tobacco (Table 3).

### Prevalence of exposure to second-hand smoke or fumes

The prevalence of second-hand smoke exposure was 36.6% (*n* = 142). Most passive smokers, 33.1% (*n* = 47), reported exposure at both home and work and 23.2% (*n* = 33) exclusively at work (see Table 3). The mean duration of second-hand smoke exposure among ‘never’ users was 13 years (s.d. = 11.9) and for ex-smokers, 7.4 years (s.d. = 8.6).

### Prevalence of factors that motivated the initiation of tobacco use

Among current tobacco users, 26.8% (*n* = 15) expressed no specific reasons for initiating tobacco use, and another 21.4% (*n* = 12) cited peer pressure. Other reported reasons shown in Table 3 include pressures from work, school and relationships, and mental health benefits.

### Quit attempts among current tobacco users

About 77% (*n* = 41) of current tobacco users had tried to quit tobacco at some stage. An estimated 56.6% (*n* = 30) indicated they had contemplated quitting in the past year, and of these, 58.5% (*n* = 24) reported that their future health concerns were the main motivating reason to try quitting. A similar response pattern was observed among ex-users. The only motivating factor for quitting that borders on statistical significance in both groups was their concern about their health. As demonstrated in Table 4, most current users 70.7% (*n* = 29) could not quit because they relied solely on their willpower.

**TABLE 4:** Factors attributable to successful and unsuccessful quit attempts among tobacco users.

Motivation to quit	Current users (*n* = 41)		Ex-users (*n* = 40)		Frequency (*n*)	Percentage (%)	*p*-value
	Frequency (*n*)	Percentage (%)	Frequency (*n*)	Percentage (%)			
**A. A comparison of current and ex-tobacco users’ motivations for quitting**							
My current health concerns	19	46.3	7	17.5	-	-	0.01
My future health concerns	24	58.5	22	55.0	-	-	0.75
Financial costs	12	29.3	7	17.5	-	-	0.21
I am aware of the risks of tobacco use	21	51.2	21	52.5	-	-	0.91
Concern for the health of my family	8	19.5	8	20.0	-	-	0.96
Advice from a healthcare provider	6	14.6	2	5.0	-	-	0.26
My tobacco use is socially unacceptable	2	4.9	2	5.0	-	-	1.00
Pressures from significant person(s)	0	0.0	4	10.0	-	-	0.06
Restriction of tobacco use at work	3	7.3	0	0.0	-	-	0.24
Restriction of tobacco use at home	0	0.0	1	2.5	-	-	0.49
Anti-tobacco campaigns	0	0.0	2	5.0	-	-	0.24
Influence of a significant person who stopped tobacco use	2	4.9	3	7.5	-	-	0.68
My social or work stressors got better	4	10	3	7.5	-	-	1.00
My desire to be more financially prudent	4	10	4	10	-	-	1.00
**B. Factors attributed to unsuccessful quit attempts among current tobacco users**							
I thought I could quit on my own (self-will only)	-	-	-	-	29	71	-
Peer pressure	-	-	-	-	5	12	-
Tobacco is a source of pleasure for me	-	-	-	-	6	15	-
I did not want to gain weight	-	-	-	-	3	17	-
My significant one(s) also use tobacco	-	-	-	-	1	2	-
Work pressures	-	-	-	-	2	5	-
Stress in my social life	-	-	-	-	13	32	-
Nicotine cravings	-	-	-	-	12	29	-
I did not have treatment support	-	-	-	-	5	12	-
I could not handle the withdrawal symptoms	-	-	-	-	2	5	-
I do not know	-	-	-	-	2	5	-

### Healthcare provider’s readiness and willingness to provide tobacco cessation treatments

About 80.5% (*n* = 354) of the respondents reported that it was important (i.e. extremely or very important) to implement TCT in their patients who use tobacco, as shown in Table 5. The study used a one-sample *t*-test to determine whether the scores above the central point of five were significantly important or not. The results showed a mean score above the central point (mean = 8.5, s.d. = 2.3, *p* < 0.001) implying that it was significantly important for respondents to implement TCTs.

**TABLE 5:** Readiness to implement tobacco cessation treatments among respondents.

Variable	Ready		Not ready		*P*-value
	Frequency	%	Frequency	%	
**Age (years)**	-	-	-	-	0.98
19–29	52	17.7	27	19.1	-
30–39	93	31.7	44	31.2	-
40–49	69	23.5	36	25.5	-
50–59	59	20.1	25	17.7	-
60+	20	6.8	9	6.4	-
Total	293	100.0	141	100.0	-
**Gender**	-	-	-	-	0.20
Female	234	79.3	121	84.6	-
Male	61	20.7	22	15.4	-
Total	295	100.0	143	100.0	-
**Marital status**	-	-	-	-	0.84
Divorced	10	3.3	8	5.6	-
Single	160	53.5	80	55.6	-
Married	95	31.8	42	29.2	-
Widowed	14	4.7	5	3.5	-
Separated	3	1.0	2	1.4	-
Cohabiting	17	5.7	7	4.9	-
Total	299	100.0	144	100.0	-
**Professional category**	-	-	-	-	0.91
Medical practitioner	54	18.0	22	15.3	-
Dental practitioner	8	2.7	4	2.8	-
Nurse clinicians	145	48.3	71	49.3	-
Community health worker	93	31.0	47	32.6	-
Total	300	100.0	144	100.0	-
**Ethnicity**	-	-	-	-	0.26
Black people	269	90.3	136	94.4	-
Mixed race people	2	0.7	1	0.7	-
Indian people	15	5.0	2	1.4	-
White people	12	4.0	5	3.5	-
Total	298	100.0	144	100.0	-
**Years of service**	-	-	-	-	0.20
1–5	81	28.3	36	25.9	-
6–10	67	23.4	33	23.7	-
11–15	66	23.1	31	22.3	-
16–20	29	10.1	15	10.8	-
21+	43	15.0	24	17.3	-
Total	286	100.0	139	100.0	-
**Tobacco use**	-	-	-	-	0.50
Never users	238	79.3	110	76.4	-
Ex-users	28	9.3	12	8.3	-
Current users	34	11.3	22	15.2	-
Total	300	100.0	144	100.0	-
**Quit attempts**	-	-	-	-	0.31
Attempted to quit	28	82.4	13	68.4	-
Not attempted to quit	6	17.6	6	31.6	-
Total	34	100.0	19	100.0	-

When asked to rate their confidence in implementing all levels of TCT, from 0 (not at all) to 10 (extremely confident) most, 56.3% (*n* = 245) felt reasonably confident. The average score of 7.13 was found to be significantly higher than the central rating score of ‘5’, *p* < 0.001, thus indicating confidence in implementing the treatments (s.d. = 2.8, CI = 1.69 – 12.58).

On a scale of 10, most respondents, 82.2% (*n* = 365), were reasonably willing to implement tobacco treatments, with a mean score of 8.27 (s.d. = 2.028). In bivariate analysis, willingness to offer tobacco did not differ across socio-demographic characteristics, tobacco use status (*p* = 0.344) or training received during clinical practice (*p* = 0.28).

An estimated 68% (*n* = 300) of respondents who achieved a minimum score of 6 out of 10 on the readiness scale were classified as ‘ready’ to implement TCTs. Readiness to implement TCTs did not differ across socio-demographic characteristics, previous training on tobacco, quit attempts or tobacco use status, as shown in Table 5. However, respondents who were classified as ready to offer TCTs were significantly more willing than those who were not, *p* < 0.001.

Concerning screening, 29.1% (*n* = 129) reported that they always screened patients for tobacco use, and 13.5% (*n* = 60) never screened. Regarding what activities they could confidently implement in TCT, most respondents could give brief advice, 62% (*n* = 276). In contrast, fewer knew how to conduct in-depth counselling, 22% (*n* = 99), and only 9% (*n* = 42) of respondents knew how to prescribe drugs for tobacco use cessation. Concerning the aspects of TCTs each category of HCPs could perform, most doctors could screen, 71.1% (*n* = 54), give brief advice, 78.9% (*n* = 60), while fewer could provide in-depth counselling, 28.9% (*n* = 22) and prescribe cessation treatments, 26.3% (*n* = 20). Among nurses, most could screen, 55.1% (*n* = 119), give brief advice, 63.9% (*n* = 138) and some could conduct in-depth counselling, 21.8% (*n* = 47) and prescribe TCTs, 5.6% (*n* = 12). Additionally, some CHWs were able to screen, 56.4% (*n* = 79), give brief advice, 49.3% (*n* = 69), offer in-depth counselling, 19.3% (*n* = 27) and prescribe TCTs, 5.7% (*n* = 8).

About 42.9% (*n* = 24) of current tobacco users reported that their tobacco use did not influence their ability to intervene in their patients’ tobacco use. When all the respondents were asked if healthcare providers who use tobacco were likely to implement TCTs, 59% (*n* = 258) reported this was less likely. However, in bivariate analysis, there were no associations between tobacco use and the ability of HCPs to screen (*p* = 0.68), give brief advice (*p* = 0.37), provide in-depth counselling to patients (*p* = 0.63) or prescribe smoking cessation drugs (*p* = 0.68).

Most of the HCPs, 66.9% (*n* = 297), did not receive training on tobacco use, its dangers and TCT during their formal training or school, and 73.4% (*n* = 326) had not received this training during their working years, either as standalone lectures, seminars or workshops. Of those who have received any training, lectures and assignments were the most frequently cited sources. In bivariate analysis, there were no associations between readiness to implement TCT and reporting previous training on tobacco use during schooling (*p* = 0.64) or working years (*p* = 0.28). However, respondents who received training at school were significantly more willing to offer tobacco cessation advice to patients who use tobacco (*p* < 0.001).

## Discussion

This study found that tobacco use is common among HCPs in primary care. Medical doctors and dentists reported to be using newer tobacco products such as electronic cigarettes, hookah and Hubble Bubble. While most current tobacco users have attempted to quit, there was no statistically significant difference between respondents’ readiness to implement TCTs and their tobacco use status. Respondents who were determined ‘ready’ to implement TCTs were significantly more willing to implement them. The outcomes of this study hold significant implications for clinical practice, policy-making and public health and underscore the importance of promoting TCTs among HCPs, providing training during and after formal healthcare education programmes and offering treatments and support to increase more quit attempts and consequently increasing quit rates among tobacco users.

This study shows lower tobacco usage in South African HCPs compared to other developing countries, China (37%) and Central and South America (25%), as shown in a 2019 review.^12^ This discrepancy is indicative of South Africa’s more progressive tobacco control programme.^32^ While previous studies on tobacco use in South Africa have primarily focussed on the general population,^5,33^ no published research has been conducted nationally on HCPs and their tobacco use to compare the findings of this study.

This study found that the prevalence of tobacco use among HCPs is lower than that previously reported in the general population (21.6% vs. 29.4%).^5^ Additionally, this study reports a reduced smoking prevalence compared to the KwaZulu-Natal study by Okeke et al. (16.2% vs. 18.1%).^20^ The latter study analysed smoking rates and patterns among all HCPs in three public hospitals. Of note, Okeke et al. could have reported a higher smoking prevalence in their sample, which comprised paramedics, administrative clerks and auxiliary HCPs with similar socioeconomic characteristics to the working class, where smoking is more prevalent.^34^ Although lower than the national average, the high prevalence of tobacco use by HCPs is an established barrier to using HCPs as front liners in the tobacco control programme as they are less likely to intervene in their own patients’ tobacco use.^13^ Healthcare professionals who use tobacco may feel a sense of cognitive dissonance when advising their patients to quit while they continue to use. This can be compounded by a fear of being perceived as hypocritical if they counsel their patients to quit tobacco.^35^ On the clinical front, the negative influence of HCPs’ tobacco use can be mitigated by helping them improve their health behaviour through a combined strategy of Screening, Brief Intervention and Referral to Treatment (SBIR/T). This technique will successfully assist individuals in modifying their risk behaviours and reduce adverse health outcomes in this critically skilled population.^36^

The prevalence of current tobacco use among CHWs (27.1%) in this study was high. This is concerning because CHWs are an excellent community-based resource for disseminating anti-tobacco information within targeted populations and liaising between patients and the health and social service providers within a community.^37^ Community health workers may have fewer resources and support for smoking cessation than doctors and nurses. Additionally, their formal training is by far limited, and so their knowledge of the risks and consequences of tobacco use may also be limited. Hence the higher prevalence of tobacco use. Furthermore, unlike nurses, dentists and doctors, CHWs may come from less privileged socioeconomic backgrounds where tobacco use is more prevalent.^34^ Irrespective of whichever explanations apply, this finding highlights the need to target all HCPs but in particular CHWs in Soweto for TCTs. This should not be only for the clinical benefit of the CHWs but by extension to facilitate their serving as health role models for the households under their care.

Regarding the intention to quit smoking, more than half of current smokers were considering quitting (56.6%), and the majority (77%) had attempted to quit in the last year. The main barrier to quitting was their reliance on willpower (70.7%). That most HCPs who are tobacco users intend to quit but rely solely on willpower emphasises the importance of providing motivational counselling to these community role models. Additionally, when necessary, it is crucial to offer pharmacotherapy, which has been proven to further increase the chances of successfully quitting.^38^ Also, relying on willpower is ineffective; only a small proportion of quitters will succeed.^39^ The goal should be to provide ongoing treatments and support during each quit attempt, as the probability of success increases with each additional attempt.^30^ However, the skill to provide motivational counselling and tobacco cessation drugs are not readily available in public healthcare services, highlighting the need for public and occupational health policy change to ensure the availability of evidence-based tobacco cessation therapies. Other interventions, such as peer support groups, face-to-face counselling and over-the-phone support through quit lines, are also recommended by the WHO as effective tobacco cessation methods.^2^ However, it appears that HCPs are unaware of these interventions’ effectiveness for tobacco cessation and so do not use them. Hence, tobacco users who want to quit may not receive adequate support, and continued tobacco use increases their risk of health issues, higher healthcare costs and lower quality of life.^40^

Many HCPs did not receive training on tobacco use and its cessation interventions during their formal education and ongoing employment. Perez et al. (2018) found that only about 36% of health sciences students in two major South African universities received formal training on tobacco use and TCTs, revealing a gap in their curricula.^18^ This could explain why the years of experience as HCPs did not influence respondents’ readiness to perform TCTs, and it further suggests that HCPs do not utilise TCTs in their patient care or the in-service training priorities. Indeed, an HCP’s continuous practice of TCTs will increase their competence.^41^ The lack of competence regarding TCTs among HCPs is an indictment of health professions training in South Africa, as tobacco-related illnesses account for a significant portion of the disease burden in the country.^42^ As health priorities and disease burdens should drive health professions training, this should also be appropriately represented in the curricula of all health-related disciplines and continued professional development. However, respondents reporting receipt of training did not influence their readiness to implement TCT in this study, possibly because of ignorance about its effectiveness. On the contrary, HCPs reported willingness to intervene was significantly influenced by the lectures they received during their school years. This is consistent with previous studies in Ethiopia and Namibia, which reported that HCPs with formal training are more willing to practice TCTs.^43^ Our study showed no relationship between formal tobacco cessation training and readiness for TCT. However, a review by Carson et al.^44^ found that HCPs who report receiving training on TCT are more likely to counsel, set a quit date and follow up with patients. Overall, the evidence suggests that well-designed and implemented training programmes can make a difference in the HCP delivery of TCTs.^22,44^ However, the research landscape is nuanced, and several other factors might have been responsible for the findings observed in this study.

Most respondents were ready and willing to implement TCTs for tobacco users, regardless of tobacco use, socio-demographics or quit attempts. Surprisingly, most tobacco users reported that their use did not influence their ability to provide TCTs. This finding is counter-intuitive and contradicts the general belief that HCPs who use tobacco are less likely to offer smoking cessation counselling to their patients because of moral conflicts.^45^ The literature is not settled on this issue, as indicated in a systematic review by Ilesanmi et al.^13^ that in Egypt, doctors reported that whether or not they smoked did not affect how they helped people quit. However, studies conducted among nurses in Croatia,^46^ physicians in the United States^14^ and dentists in India^47^ all reported that HCPs who use tobacco were less likely to implement TCTs in their patients. The unsettled evidence in this regard may indicate that the concept of willingness and readiness to implement TCT, though measured similarly, is a complex behaviour, and many factors other than HCP’s training status may influence it. Hence, further studies may be required to fully understand HCPs’ behaviours properly. Of note is that the sample in our study is mostly comprised of nurses, which could have skewed the findings in favour of nurses’ opinions. However, nurses are the largest category of HCPs in South African primary healthcare, and to the extent that this is true, the study findings remain generalisable in this context.

### Strengths and limitations

This study provides insight into how HCPs’ tobacco use status influences their readiness to implement tobacco cessation interventions in South Africa. The findings have important implications for clinical practice and policy, but there are limitations to consider for future studies. Firstly, the study was not a national study, and differences in the composition of HCPs may result in different patterns and prevalence of tobacco use, particularly as some socio-demographic characteristics may vary across settings and regions. For example, the underrepresentation of some ethnic groups may have led to an underestimation of the prevalence of tobacco use, as mixed race, white and indian populations typically have higher smoking rates compared to a nearly homogenously black population as in Soweto. Nonetheless, the study recruited the entire target population in the subregion, and its sample was, therefore, representative of what applies in the five community health centres (CHC). Secondly, the study relied on self-reports and lends itself to recall and social desirability biases. These could have influenced HCPs to under-report their smoking habits for fear of judgement as they are usually regarded as role models. To limit this, the study created a non-judgmental environment that enabled respondents to report their tobacco use and habits truthfully. Finally, the cross-sectional design limits the generalisability of the study findings and cannot establish any causal relationships between the variables.

## Conclusion

This study found that the prevalence of tobacco use among HCPs in South African primary health care is high, and most of these professionals are making efforts to quit. These findings call for tobacco cessation programmes that screen for tobacco use among HCPs and motivate and support users in this population to quit. Although most HCPs were willing and/or ready to implement TCTs to varying degrees, there remains a need to scale up training on tobacco use and treatment for HCPs and integrate TCTs into routine services in primary healthcare. The unsettled evidence on how tobacco use status affects HCPs’ willingness and/or readiness to implement TCT calls for further studies utilising more rigorous designs and methods and more nationally representative samples of HCPs.
